# Enzymatic Extraction, Purification, and Characterization of Polysaccharides from* Penthorum chinense Pursh*: Natural Antioxidant and Anti-Inflammatory

**DOI:** 10.1155/2018/3486864

**Published:** 2018-11-27

**Authors:** Li-mei Lin, Ling-jia Zhao, Jing Deng, Su-hui Xiong, Jie Tang, Ya-mei Li, Bo-hou Xia, Duan-fang Liao

**Affiliations:** ^1^College of Pharmacy, Hunan University of Chinese Medicine, Changsha 410208, China; ^2^Collaborative Innovation Center for the Protection and Utilization of Chinese Herbal Medicine Resources in Hunan Province, Hunan University of Chinese Medicine, Changsha 410208, China

## Abstract

*Penthorum chinense Pursh* (PCP) is a kind of functional food or medicine for liver protection. In the present work, Plackett-Burman design, steepest ascent method, and response surface methodology (RSM) were employed to obtain maximum total sugar yield. The experimental yield of 6.91% indicated a close agreement with the predicted yield of 7.00% of the model under optimized conditions. The major polysaccharide fraction (PCPP-1a) from PCPP was purified and identified as acidic polysaccharides with a high content of uronic acid (FT-IR, UV, HPGPC). PCPP had similar monosaccharide profile with PCPP-1a but was rich in galacturonic acid (HPLC). Both of PCPP and PCPP-1a possessed strong hydroxyl radical scavenging, DPPH radical scavenging, and Fe^2+^ chelating activities. Moreover, they were revealed to show strong anti-inflammatory activities by inhibiting NO, TNF-*α*, and IL-1*β* release compared to LPS treatment in RAW264.7 cells. These data suggest that the polysaccharides from PCP could be potential natural products for treating ROS and inflammatory-related diseases.

## 1. Introduction

Polysaccharide, a crucial biomacromolecule in living organisms, is one of the most abundant storage carbohydrates of plants [1]. Botanical polysaccharides were involved in multiple bioactivities such as antioxidation, immunomodulation, antidiabetic, and anticancer [2–5]. Moreover, botanical polysaccharides have attracted increasing attention for their health-related benefits and therapeutic use with relatively high security [6, 7]. Therefore, they have immense potential industrial applications in pharmaceuticals [8], cosmetics [9], foods [10], and materials [11].

*Penthorum chinense Pursh* (PCP, Saxifragaceae) mostly distributed in China (Miao ethnomedicine), eastern Russia, Japan, and Korea [12–16]. It has been used traditionally as functional food or traditional Chinese medicine for treating liver disease [17, 18]. Recently, several studies indicated that PCP exhibited antioxidant, hypoglycemic, antiproliferative, and anticomplement activities [19–22]. According to the preparation process of previous studies, the water-soluble constituents were the main focus [23]. Among them, flavonoids, flavonoid glycosides, and other phenolic contents accounted for the major constituents [19, 22, 24]. Unfortunately, studies on the extraction, characterization, and bioactivities of* Penthorum chinense* Pursh polysaccharides (PCPP) are limited.

Accordingly, this study was first designed to optimize the extraction process of PCPP using design of experiment (DOE). The crude PCPP was then purified by DEAE-52 cellulose and Sephadex G-150. Moreover, chemical analysis (HPLC, HPGPC, and FT-IR), antioxidant activities, and anti-inflammatory effects of PCPP and its purified fractions were further characterized.

## 2. Materials and Methods

### 2.1. Materials

PCP was acquired from Gaoqiao natural herbal special market (Changsha, China). The dried PCP sample was pulverized by a disintegrator (HX-200A, China) and passed through an 80-mesh screen (Harbin Ouerfu Filter Material Co., Ltd., China) to obtain the final powder.

3-(4,5-Dimethylthiazol-2-yl)-2,5-diphenyltetrazolium bromide (MTT), Dextrans of different molecular weights, 2,2-diphenyl-1-picrylhydrazyl (DPPH), and lipopolysaccharide (LPS) were obtained from Sigma Chemical Co. (St. Louis, MO, USA). Total antioxidant capacity assay kit (ABTS, FRAP) was obtained from Beyotime Biotechnology (Jiangsu, China). Dulbecco's Modified Eagle medium (DMEM) and fetal bovine serum (FBS) were acquired from Gibco (Grand Island, NY, USA). DEAE-52, Sephadex G-150, monosaccharide standards (glucose (Glc), galactose (Gal), ribose (Rib), mannose (Man), glucuronic acid (GlcUA), rhamnose (Rha), xylose (Xyl), galacturonic acid (GalUA), arabinose (Ara), and fucose (Fuc)), and cellulase were acquired from Sinopharm Chemical Reagent Co., Ltd.

### 2.2. Preparation of PCP and Determination of the Yield

After drying to a constant weight and homogenization, PCP powder was extracted three times with 80% ethanol at 60°C, each for 5 h, to remove lipids, amino acids, monosaccharide, oligosaccharides, and some colored materials [25]. Pretreated samples were centrifuged (4000 rpm, 20 min), and the powder was vacuum-dried to a constant weight. Each dried and pretreated sample (5 g) was extracted by ultrasound-assisted enzymatic (cellulase) extraction method at the designated extraction conditions. Extraction conditions were controlled by varying the ratio of liquid-to-solid, enzyme concentration, pH value, ultrasonic power, time, and temperature. Extracts were centrifuged at 5000 rpm for 10 min. The supernatant was merged and precipitated by overnight incubation with ethanol (80%; v/v; final concentration). The precipitation was harvested by centrifugation (5000 rpm, 20 min) and lyophilized at -50°C to acquire crude polysaccharides. The percentage of the yield of polysaccharides (%) was estimated using the formula *y*(%) = *w*_1_/*w*_0_ × 100%, where* Y* was the PCPP extraction yield (%), *w*_1_ was the polysaccharides of extraction (g), and *w*_0_ represented dried sample weight (g).

### 2.3. Plackett-Burman Screening Designs

Plackett-Burman design (PBD) is a highly fractional design which identifies the significant factors from a multivariable system and considerably diminishes the number of experiments [26]. This technique has been widely used to screen the significant factors prior to optimization [27]. A two-level (high and low) PBD was tested for 6 selected factors (Table 1). Minitab 16.0 (Minitab Inc., USA) was applied to create the design matrix shown in Table 2. The yield of PCPP was analyzed in triplicate for each test and the averages were regarded as the responses.

### 2.4. Steepest Ascent Method

Steepest ascent method based on steepest ascent lines was applied to get rapidly to the neighborhood of the optimum. This way established an effective path through the center of the design based on the results from the PBD [28].

In the present work, tests with defined intervals for each response were conducted by the path of steepest ascent. The obtained design and results are displayed in Table 3. Once there is no further achieved improvement in the response, the point would be the general vicinity of the optimum and could be taken as the center point of central composite design (CCD).

### 2.5. Response Surface Methodology (RSM)

In order to understand the interactions among the factors and optimize the extraction conditions for PCPP, RSM via central composite design (CCD), which includes various processes such as regression analysis and factorial design was applied [29]. In this study, a CCD experimental plan with two variables and three levels was utilized to get the optimal conditions for PCPP. The three levels of the significant factors (enzyme concentration and liquid-to-solid ratio) were set as -1, 0, and 1 for high, intermediate, and low values, respectively (*α*=1.414). The experiments were executed based on the matrix established by the Design-Expert soft (Version 10). The design matrix with coded and actual values of the factor for 13 experiments which were constructed with 5 central point replications is manifested in Table 4. Once the experiments had been conducted, the PCPP yield (Response variable) was fitted with a second-order model. The basic form of the equation appears as follows:

(1) $$\begin{array}{c} Y=\beta_{0}+\overset{k}{\underset{i=1}{\sum }}\beta_{i}x_{i}+\overset{k}{\underset{i=1}{\sum }}\beta_{ii}{x_{i}}^{2}+\sum_{i=1}\sum_{j=i+1}\beta_{ij}x_{i}x_{j}+\varepsilon \end{array}$$

where* Y* is the predicted response (PCPP yield), *β*_0_ is the constant term, *β*_*i*_, *β*_*ii*_, and *β*_*ij*_ are the regression term coefficient for the linear, quadratic, and interaction effect, respectively. *χ*_*i*_ and *χ*_*j*_ refer to the actual levels of the design variables (independent variables) and *ε* is the error.

### 2.6. Fraction and Purification of Crude PCPP

The crude PCPP was dissolved in distilled water and subjected to a DEAE-52 cellulose column (500 mm × 30 mm) and then washed sequentially with distilled water, 0.1, 0.3, and 0.5 M NaCl solutions, and 0.5 M NaOH solution at a flow rate of 1.0 mL min^−1^. Four separated fraction peaks were obtained by checking the absorbance at 490 nm according to the phenol-sulfuric acid assay [30]. The four peaks were concentrated and precipitated using four times 100% (v/v) ethanol overnight at 4°C. The precipitation was then yielded by centrifugation and subsequently lyophilized. The major fraction, designated as PCPP-1, was further applied to Sephadex G-150 (800 mm × 30 mm) and eluted with NaCl solution at a flow rate of 0.4 mL min^−1^. As a result, a major purified fraction was harvested, dialyzed, and freeze-dried, affording PCPP-1a.

### 2.7. Analysis of Polysaccharides Characterization

#### 2.7.1. Determination of Carbohydrate, Sulfuric Radical, Uronic Acid, Protein, and Total Polyphenol

The contents of carbohydrate in crude PCPP and its purified fraction (PCPP-1a) were evaluated by the phenol-sulfuric acid assay using a series of concentrations of D-glucose as standards [31]. The content of sulfate radical was assessed using the reported way [32]. The uronic acid content of PCPP and PCPP-1a was measured by m-hydroxydiphenyl method using D-galacturonic acid as a standard [33]. The protein content was measured conforming to the Bradford method using bovine serum albumin (BSA) as a standard [34]. The total polyphenols content was estimated by the Folin–Ciocalteu colorimetric method reported by Singleton and Rossi [35], and the result was given as mg GA equivalent (GAE) per mg dry sample (mg GAE /100 mg dried extract).

#### 2.7.2. Analysis of Molecular Weight (Mw) and Monosaccharide Composition

High-performance gel-permeation chromatography (HPGPC) equipped with a column (7.8 mm × 300 mm, 5 *μ*m) and a RID-10A refractive index detector was applied to detect the Mw of the fraction of PCPP [36]. The column was eluted with 0.002 M NaH2PO4 solution (contains 0.05% NaN3) at a flow rate of 0.6 mL.min^−1^. The Mw was determined by a calibration curve using a set of standard Dextran with different molecular weights.

Monosaccharides compositions of polysaccharides were determined by 1-phenyl-3-methyl-5-pyrazolone (PMP) derivation way using high-performance liquid chromatography (HPLC) [37]. The standard monosaccharides included mannose (Man), galactose (Gal), ribose (Rib), rhamnose (Rha), xylose (Xyl), arabinose (Ara), glucuronic acid (GlcUA), galacturonic acid (GalUA), glucose (Glc), and fucose (Fuc). These standard monosaccharides were also derived in the same manner and applied to analysis. The HPLC was operated using Kromasil C18 Column (250 mm × 4.6 mm, 5 *μ*m, Akzonobel, Sweden) at 30°C lasting 60 min at a flow rate of 1 mL.min^−1^. A mixture of phosphate buffered saline (PBS, 0.1 M, pH 6.8) (solvent A) and acetonitrile (solvent B) was taken as the mobile phase. The elution concentration gradient of solvent B was 16%, 16%, 18%, 19%, and 19% corresponding to 0, 10, 30, 50, and 60 min, respectively. The injection volume was 10 *μ*L.

#### 2.7.3. FT-IR Spectrometric Analysis

The structures of crude PCPP and its purified fraction (PCPP-1a) were analyzed by Attenuated Total Reflectance Fourier Transformed Infrared Spectroscopy (ATR-FTIR). Spectra were recorded on a Nicolet iS5 spectrometer, equipped with an iD5 ATR accessory (Thermo Scientific, USA), detecting in transmittance mode with a resolution of 4 cm^−1^, a frequency range of 4000-525 cm^−1^, and 64 scans.

### 2.8. Antioxidant Activity

#### 2.8.1. ABTS^+^ Radical Scavenging Activity

The radical scavenging activities of crude PCPP and PCPP-1a against ABTS^+^ were measured by an ABTS assay kit using the method in line with the manufacturer's instructions. Absorbance was measured at 414 nm using a microplate reader in a Multimode Microplate Reader (Thermo Scientific, USA), with Trolox as an antioxidant standard. The antioxidant activity of the sample was calculated using

(2) $$\begin{array}{c} ABTS\;\;scavenging\;\;effect\;\left(\;\%\;\right)=\left(\;A_{B}-A_{A}\;\right)\times \frac{100}{A_{B}} \end{array}$$

where *A*_*B*_ and *A*_*A*_ represent the absorbance values of the blank and of the tested samples, respectively.

#### 2.8.2. Ferric-Reducing Antioxidant Power (FRAP)

The determination of FRAP was performed to measure the total antioxidant capacity of crude PCPP and PCPP-1a using a commercially available assay kit in a Multimode Microplate Reader (Varioskan Flash, Thermo Scientific, USA) referring to the manufacturer's instructions. The results were calculated by using the linear calibration curve and presented as per g of dry weight (mM FeSO_4_/g dried extract). The above experiment was performed three times.

#### 2.8.3. DPPH Scavenging Assay

The DPPH radical scavenging activity was examined using the following Vallverdú-Queralt et al. method with slight modifications [38]. Solutions of known Trolox concentrations were taken as calibration. 20 *μ*L of samples or Trolox were mixed with 200 *μ*L DPPH (DMSO, 0.1 mg mL^−1^). Absorption of the mixture was measured after the homogenate was shaken and incubated in darkness for 30 min.

(3) $$\begin{array}{c} Inhibition\%=\frac{A_{0}-A}{A_{0}}\times 100\% \end{array}$$

### 2.9. Anti-Inflammatory Activity

#### 2.9.1. Cell Culture and Viability Assay

RAW264.7 macrophages were obtained from the Cell Line Bank of Central South University (Changsha, China) and maintained in DMEM medium supplemented with 10% (v/v) fetal bovine serum, as well as 1% penicillin and streptomycin at 37°C in a humidified atmosphere with 5% CO_2_. The toxicity profile of crude PCPP and PCPP-1a was assessed using the MTT viability assay according to a previous study [39].

#### 2.9.2. Quantification of NO, TNF-*α*, and IL-1*β*

The anti-inflammatory activity was determined according to a previous method [40]. RAW 264.7 macrophages were pretreated with 0, 15, 30, 60, and 120 *μ*g.mL^−1^ of crude PCPP and PCPP-1a for 24 h and washed before challenging with LPS (1 *μ*g mL^−1^). The cells cultured in DMEM with the absence of polysaccharides and LPS were used as the normal control. The 24h LPS-stimulated proinflammatory mediator and cytokine production were observed by detecting NO, TNF-*α*, and IL-1*β* levels in the culture medium using commercial kits (Becton Dickinson Medical Devices Co. Ltd., China) according to the instruction of the manufacturers. All the experiments were implemented in triplicate.

### 2.10. Statistical Analysis

Results were given as means ± SD of three replicated for each test and analyzed by SPSS 18.0 software (SPSS, USA). Statistical differences of data were analyzed by one-way ANOVA, followed by least significant difference test (LSD-t). Statistical significance was set at* p *<0.05.

## 3. Results and Discussions

### 3.1. Screening Test of Plackett-Burman Design

Six selected independent factors on PCPP yield were investigated using PBD. As shown in Table 2, PCPP yield among the 12 experimental tests indicated a substantial variation, going from 0.64% to 4.23%, suggesting a great influence of the screened parameters on PCPP extraction yield. The* P* value is defined as the probability for a given model in the statistical hypothesis testing [41]. A* P* value less than alpha of 0.05 was considered statistically significant. According to the ANOVA analysis of PBD (Table 2), the liquid-to-solid ratio (*p*=0.015) and enzyme concentration (*p*=0.001) made significant influences on PCPP yield. In contrast, extraction temperature (*p*=0.149), extraction time (*p*=0.529), ultrasonic power (*p*=0.333), and pH (p=0.833) showed no statistically significant effect within the considered range. Moreover, the determination coefficient (*R*^2^) was used to evaluate the goodness of fit of the regression model [42]. *R*^2^ of PCPP yield was found to be 0.9332, which showed a goodness of the model, explaining 93.32% of the variability of the response. Therefore, the variables, liquid-to-solid ratio (*X*_3_), and enzyme concentration (*X*_5_), with significant influences, were included in the path of steepest ascent.

### 3.2. The Path of Steepest Ascent

The path of steepest ascent was utilized to increase liquid-to-solid ratio (*X*_3_) and concentration of enzyme (*X*_5_) with step sizes of 10 and 1, respectively, in order to get the central point of the optimum. When continuing to increase the value of* X*_3_ and* X*_5_, there is no further improvement in the response, suggesting that it was the neighborhood of maximum response (PCPP). As shown in Table 3, among the five tested experiments, PCPP yield attained maximum value when* X*_3_ and* X*_5_ increased to 20 mL.g^−1^ and 5%, respectively. Therefore, the levels of 20 mL.g^−1^ and 5% were set as the center point of CCD.

### 3.3. Optimization by Response Surface Methodology (RSM)

Once the center point of the experiment was defined, RSM via CCD was applied to fine-tune the two significant factors. Multiple regression analysis was applied to establish the mathematical relation between the experimental factors and PCPP yield. By using this method, a second-order polynomial model for PCPP yield is presented as follows:

(4) Y=−38.50872+14.63311∗x5+0.25229∗x3+0.030904∗x3∗x5−1.33140∗x52−1.33140∗x32

where* Y* (%) is the predicted PCPP yield,* X*_3_ is liquid-to-solid ratio, and* X*_5_ is enzyme concentration.

The second-order model obtained from the multiple regression analysis is analyzed and the results are presented in Table 5. The F-test result of the quadratic regression model indicated a high significance (*p*<0.05), while it also showed that all regression coefficients are significant. The fitness of the polynomial model was verified through the lack of fit test. In this study, the lack of fit F value of 0.86 inferred that the model was statistically insignificant (*p* = 0.5305), suggesting that it was sufficient to predict PCPP yield within the tested range of variables. The same result was shown by model* R*^2^ (*R*^2^ = 0.9960), which indicated that the models are good fitness and less variation. The adjusted determination coefficient (*R*^2^_*adj*_ = 0.9932) was also content to confirm the model significance. The adequate precision (53.58) obtained by dividing the difference between the predicted maximum and minimum responses indicated a good signal to noise ratio (a ratio greater than 4 is desirable). These results inferred that the polynomial model successfully described the variability of the PCPP yield by the experimental factors.

**Figure 1:**
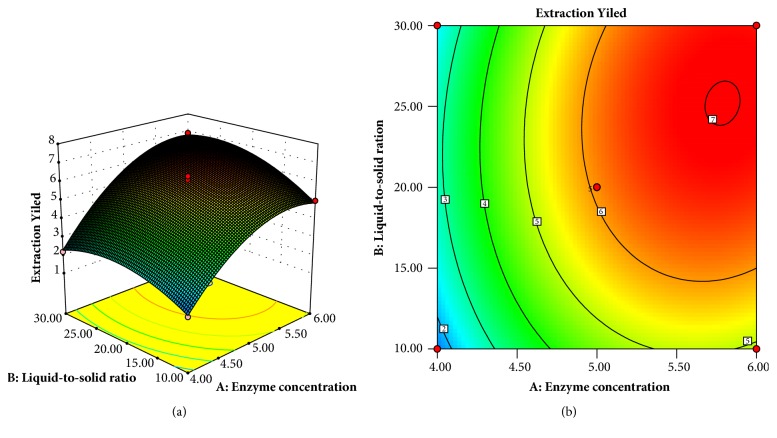
Plot (a) and contour plots (b) showing the mutual effect of liquid-to-solid ratio and enzyme concentration on the yield of PCPP.

The 3D response surface graphs and contour plots supplied a more complete representation of the regression equation (Figure 1). As shown in Figure 1(a), the PCPP yield rose as liquid-to-solid ratio and enzyme concentration increased to optimum values and then declined with further increases of them. The 2D contour plot (Figure 1(b)) showed a clearly elliptical contour, suggesting a significant interaction between *X*_3_ and *X*_5_ on PCPP yield

According to (1), the optimum conditions of the variables for the maximum PCPP yield were liquid-to-solid ratio of 26.40 mL.g^−1^ and enzyme concentration of 5.86%. Under these conditions, the maximum predicted PCPP yield was 7.00%. The prediction of the model was verified by performing a validation experiment in triplicate tests. The observed experimental PCPP yield was 6.91% (n = 3), which was very close to the predicted PCPP yield. This result proved the validity of the model.

### 3.4. Fraction and Purification of Polysaccharides

**Figure 2:**
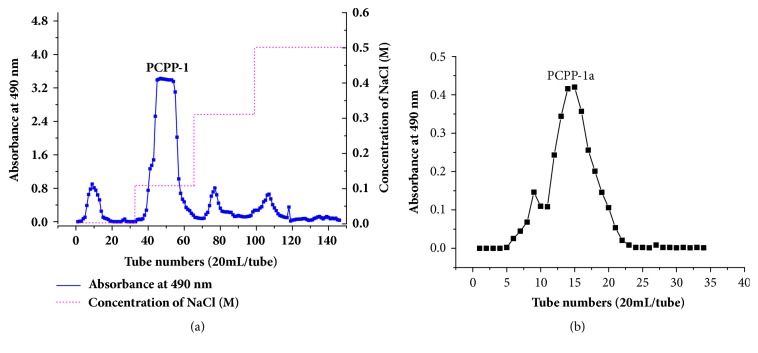
Isolation and purification of polysaccharides. The crude PCPP was further separated by DEAE-52 cellulose into several fractions, (a); the main peak (PCPP-1) was further fractionated over Sephadex-G150 column and collected main component, namely, PCPP-1a (b).

The crude PCPP extract was loaded onto DEAE-52 cellulose and then was isolated and purified according to their different ionic groups [43]. Several fractions were separated by successive elution with NaCl solutions (0, 0.1, 0.3, and 0.5 M) (Figure 2(a)). Considering the low yields of other fractions (accounted for approximately 5% of the total), only PCPP-1 was further purified by gel-filtration on Sephadex G-150 and then concentrated, dialyzed, and lyophilized to afford PCPP-1a (Figure 2(b)).

### 3.5. Preliminary Characterization of Polysaccharides

The physicochemical properties of crude PCPP and PCPP-1a were presented in Table 6. Generally, both crude PCPP and its purified polysaccharides (PCPP-1a) contained high levels of total carbohydrates (58.42 ± 5.17% for crude PCPP and 80.49 ± 6.95% for PCPP-1a) and uronic acid (42.97 ± 3.11% for crude PCPP, 21.08 ± 1.96% for PCPP-1a), suggesting that crude PCPP and PCPP-1a were acid polysaccharides [44]. No protein and a small number of total polyphenols (0.44±0.02) were detected in PCPP-1a, which indicated that the combination of DEAE-52 and Sephadex G-150 was an efficient and useful tool to isolate and purify crude PCPP. The low content of sulfuric radical (3.38 ± 0.11% for crude PCPP and 1.74 ± 0.02% for PCPP-1a) was detected in polysaccharides, respectively.

### 3.6. Chemical Characterization of Polysaccharides

**Figure 3:**
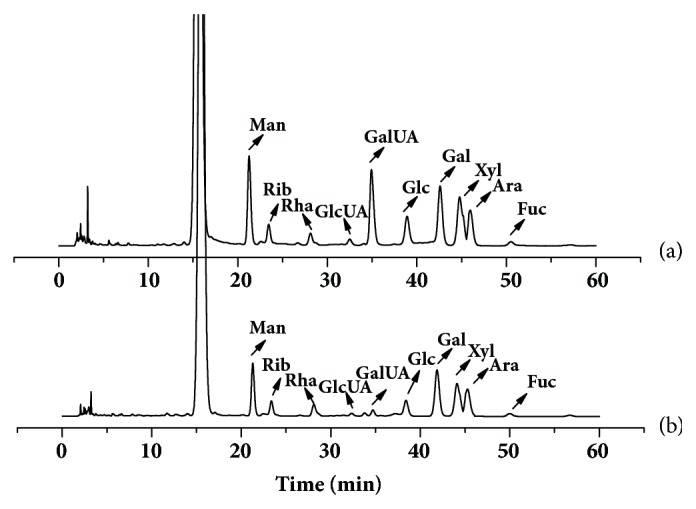
The HPLC chromatograms of monosaccharides released from PCPP (a) and PCPP-1a (b) compared with standard monosaccharides. The PMP-labeled monosaccharides were separated and identified by HPLC-UV at 250 nm (Man, mannose; Rib ribose; Rha, rhamnose; GlcUA, glucuronic acid; GalUA, galacturonic acid; Glc, glucose; Gal, galactose; Xyl, xylose; Ara, arabinose; Fuc, fucose).

As shown in Table 6, the result of the HPGPC analysis indicated that the Mw of PCPP-1a was 47.3 kDa. Also, the proportions and species of monosaccharides in both crude and fractional polysaccharides were observed (Figure 3). According to the data presented in Table 6, crude PCPP was composed of Man, Rib, Rha, GlcUA, GalUA, Glc, Gal, Xyl, Ara, and Fuc in the molar ratio of 16.61%, 5.75%, 3.86%, 2.68%, 20.96%, 8.11%, 16.05%, 14.73%, 8.20%, and 3.0%, respectively. The monosaccharide profile of PCPP-1a was a little bit different to that of PCPP, and it mainly consisted of Man, Glc, Gal, Xyl, and Ara with their corresponding mole percentages of 16.59%, 7.51%, 24.00%, 18.45%, and 12.36%, respectively, while GlcUA, GalUA, and Fuc were presented in few amounts. The results showed that the monosaccharide compositions of PCPP were more complicated than that of PCPP-1a and implied that crude PCPP was the typically acidic polysaccharide rich in galacturonic acid.

**Figure 4:**
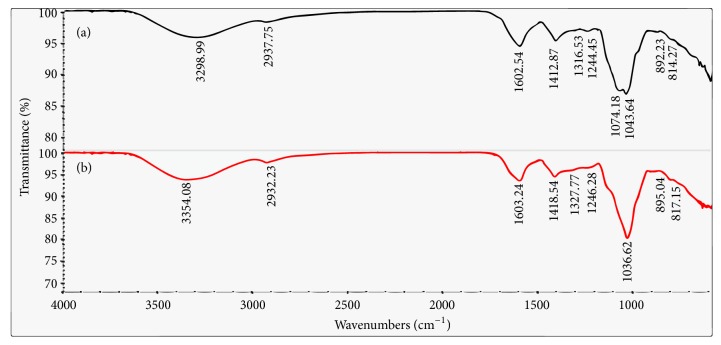
FT-IR spectrum of PCPP (a) and PCPP-1a (b) produced by PCP.

Furthermore, the measurement of functional groups was qualitatively indicated by FT-IR spectroscopy analyses [45]. As shown in Figure 4, both the crude PCPP and PCPP-1a showed typical absorption peaks of polysaccharides. A broad and intense peak nearby 3300 cm^−1^ was due to the OH stretching vibration. The bands centered around 2935 cm^−1^ were the characteristic absorption of antisymmetrical C-H stretching vibration. The absorption peaks at 1602 cm^−1^ and 1412 cm^−1^ for PCPP and 1603 cm^−1^ and 1418 cm^−1^ for PCPP-1a were attributed to the stretching vibrations of carboxylic groups (COO-) and ester carbonyl groups (C=O), respectively, suggesting that crude PCPP and PCPP-1a were acidic polysaccharides [46]. The absorbance at 1316 and 1327 cm^−1^ was due to the existence of sulfate radical [47]. The absorbance of the asymmetric stretching of ester sulfate band (S=O) nearby 1244 cm^−1^ indicated that there was content of sulfuric radical in crude PCPP and PCPP-1a [48]. The strong absorption band nearby 1043 cm^−1^ was attributed to the presence of C-O-C and C-O-H stretching vibration, which was of pyranose-ring [49]. Moreover, the perks at 816 cm^−1^ were ascribed to the characteristic absorption of mannose [50].

### 3.7. Antioxidant Activity In Vitro

**Figure 5:**
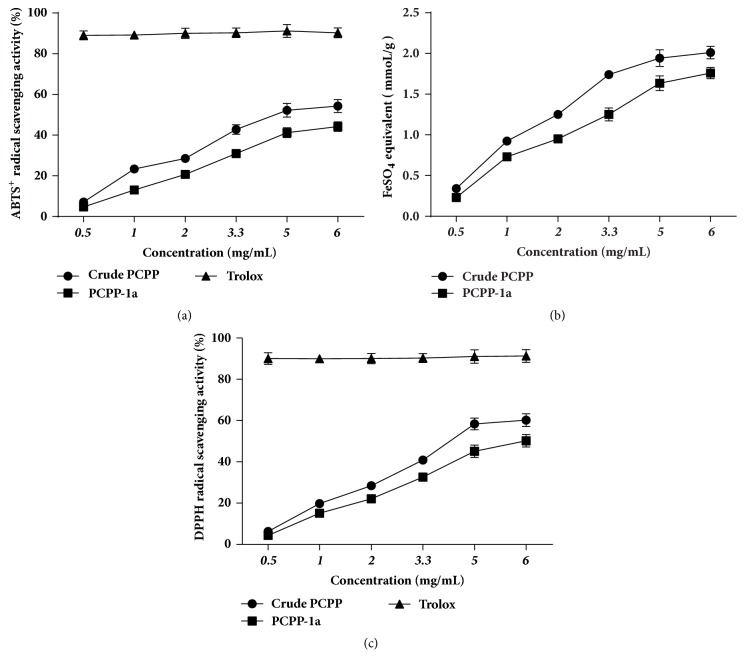
Antioxidant activity of polysaccharides. ABTS radical scavenging activity of PCPP and PCPP-1a compared with that of Trolox (a). Ferric-reducing antioxidant power of PCPP and PCPP-1a (b). Scavenging effect of PCPP and PCPP-1a on DPPH radicals compared with that of Trolox (c).

The antioxidant activities of the PCPP and PCPP-1a were determined by the ABTS^+^, FRAP, and DPPH tests. As shown in Figure 5(a), the result illustrated that Trolox, PCPP, and PCPP-1a exerted concentration-dependent effects. The scavenging ability of PCPP was relatively stronger than that of PCPP-1a across the given concentration range of 0.5-6.0 mg mL^−1^. The inhibition ratios generated by 6.0 mg mL^−1^ of PCPP and PCPP-1a were referred to be 54.32% and 44.23%, respectively, while Trolox always showed higher ABTS^+^ radical scavenging activity (>90%).

As shown in Figure 5(b), PCPP and PCPP-1a indicated different ferric-reducing antioxidant activities. The FRAP values of PCPP significantly increased in a concentration-dependent pattern and were stronger than that of PCPP-1a at each concentration point. At the highest concentration (6.0 mg.mL^−1^), the FRAP value of PCPP and PCPP-1a on the DPPH radical was 2.01 and 1.76 (mm FeSO_4_/g dried extract), respectively.

The DPPH scavenging activities of PCPP and PCPP-1a are shown in Figure 5(c) using Trolox as a positive control. In the test dosage range, the maximum DPPH radical scavenging rate of PCPP and PCPP-1a was about 60.23% and 50.23%, respectively, when its concentration reached 6.0 mg.mL^−1^; by contrast, the maximum DPPH radical scavenging rate of Trolox reached nearly 92% at the same concentration.

The superoxide radicals are toxic agents generated by numerous biological and photochemical reactions [51]. In the present study, PCPP and PCPP-1a showed the high scavenging ability of superoxide radicals. Meanwhile, because of a positive and strong correlation among the FRAP, ABTS+, and DPPH assays, a similar variation tendency was found in FRAP and DPPH assays [52]. Moreover, the mechanism of polysaccharides on radicals scavenging activities was related to the hydrogen atoms or donating electrons, and the hydrogen-donating ability was affected obviously by the differences of in monosaccharide composition, molecular weight, and conformation [53]. In the present study, the high inhibition values of PCPP compared to PCPP-1a may be due to its strong hydrogen-donating ability. According to the above, it is noteworthy that the high antioxidant properties of PCPP and PCPP-1a reported in the present study will provide an experimental evidence for the folkloric uses of* Penthorum chinense Pursh* as a good source of natural antioxidants, when considering the green extraction process of polysaccharides and inexpensive source of* Penthorum chinense Pursh*.

### 3.8. Examination of Cytokines Related to Inflammation in RAW264.7 Macrophages

**Figure 6:**
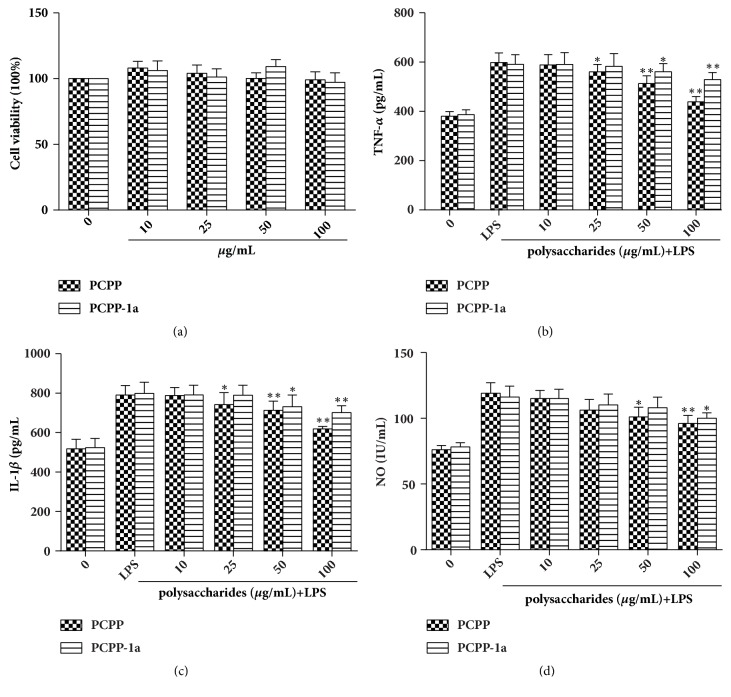
Effects of PCPP and PCPP-1a on cell viability and proinflammatory cytokines and NO production in murine macrophages. RAW264.7 cells were incubated for 24 h with increasing concentrations of PCPP and PCPP-1a and then cytotoxicity was determined by MTT assay (a). The levels of TNF-*α* (b), IL-1*β* (c), and NO (d) in the culture supernatants were determined by ELISA assay. Values are mean ± SD of three independent experiments. *∗∗p*<0.01 and *∗p* <0.05.

To calculate possible cytotoxicities of PCPP and PCPP-1a on RAW264.7 macrophages, the cells were incubated with PCPP and PCPP-1a at the concentrations of 0-100 *μ*g.mL^−1^ for 24h, respectively. The results indicated that PCPP and PCPP-1a at the tested concentrations of 10, 25, 50, 100, and 100 *μ*g.mL^−1^ (Figure 6(a)) showed insignificant influence on RAW264.7 cells viability (p>0.05). Therefore, PCPP and PCPP-1a at the tested concentrations were selected for the following tests.

As shown in Figures 6(b)-6(c), the treatment of LPS stimulated the significantly increased production of TNF-*α* and IL-1*β* in RAW264.7 cells. Meanwhile, PCPP and PCPP-1a treatments significantly inhibited the LPS-induced release levels of TNF-*α* and IL-1*β* in a concentration-dependent manner (p<0.05). Importantly, when the pretreatment of RAW 264.7 cells reached 100 *μ*g.mL^−1^ of PCPP, the production of TNF-*α* and IL-1*β* was decreased by 72.98% and 62.87%, respectively. In addition, a similar result was seen in NO production. As shown in Figure 6(d), although both PCPP and PCPP-1a show a concentration-dependent inhibition manner on NO production, they had a significant NO inhibitory effect on LPS-induced RAW264.7 cells until the concentrations reached 100 *μ*g.mL^−1^ (p<0.05 for PCPP-1a, p<0.01 for PCPP).

Macrophages, as the first defense line of the body, play a vital role in anti-inflammatory activities and immune reactions through the release of cytokines [54]. In the present study, the results indicated that PCPP and PCPP-1a were more sensitive to TNF-*α* and IL-1*β* release than NO release. Therefore, our findings suggested that PCPP and PCPP-1a may have a potential and strong anti-inflammatory against LPS-induced inflammation through reducing TNF-*α* and IL-1*β* production levels at the indicated appropriate concentrations.

## 4. Conclusions

In this work, PCPP was prepared from PCP by ultrasound-assisted enzymatic extraction, and its extraction conditions were optimized by RSM. The liquid-to-solid ratio and concentration of enzyme were defined to be the main factors. The optimum extraction conditions were liquid-to-solid ratio of 26.40 mL.g^−1^ and enzyme concentration of 5.86% to obtain the maximum yield of PCPP (6.91%). The main fraction (PCPP-1a) was further purified from PCPP. The molecular weight of PCPP-1a was 47.3 KDa. The chemical analysis results along with the FI-IR spectra implied that PCPP and PCPP-1a were acidic polysaccharides and composed of Man, Rib, Rha, GlcUA, GalUA, Glc, Gal, Xyl, Ara, and Fuc. PCPP contained more galacturonic acid than PCPP-1a. Furthermore, PCPP and PCPP-1a showed strong antioxidant and anti-inflammatory properties in vitro assays, which may provide further insights into the development and application of polysaccharides from PCP in treating ROS and inflammatory-related diseases like atherosclerosis, cardiovascular diseases, and so on. Further studies* in vivo *are needed to validate its biological functions.

## Figures and Tables

**Table 1:** Experimental field definition for the Plackett-Burman design.

**Symbol code**	**Factors**	**Experimental values**	
		**Low level (-1)**	**High level (+1)**
*X*_1_ (°C)	Extraction temperature	30	60
*X*_2_ (min)	Extraction Time	10	30
*X*_3_ (ml/g)	Liquid-to-solid ratio	10	30
*X*_4_ (W)	Ultrasonic power	200	400
*X*_5_ (%)	Enzyme concentration	2	4
*X*_6_	pH	4	5

**Table 2:** The Placket-Burman design variables (in coded levels) with PCPP yield as response.

**Run**	**Variable levels**						**Yield (** $\bar{\%}$ **)**
	*X*_1_	*X*_2_	*X*_3_	*X*_4_	*X*_5_	*X*_6_	
	(°C)	(min)	(mL/g)	(W)	(%)		
1	-1	-1	-1	-1	-1	-1	0.97
2	-1	1	1	-1	1	-1	2.81
3	-1	1	1	1	-1	1	1.55
4	1	1	-1	1	-1	-1	1.35
5	1	-1	1	-1	-1	-1	1.91
6	1	1	-1	1	1	-1	3.02
7	1	1	1	-1	1	1	4.23
8	-1	-1	-1	1	1	1	2.61
9	-1	1	-1	-1	-1	1	0.64
10	1	-1	-1	-1	1	1	2.75
11	-1	-1	1	1	1	-1	4.23
12	1	-1	1	1	-1	1	2.17
Effect	0.4353	-0.1729	0.9265	0.2737	1.8454	-0.0567	
Coefficient	0.2177	-0.0865	0.4633	0.1368	0.9227	-0.0283	
*t*-Value	1.70	-0.68	3.62	1.07	7.22	-0.22	
*p*-value	0.149	0.529	0.015*∗*	0.333	0.001*∗*	0.833	

Press = 5.65, *R*^2^ = 0.9332, and *adj-R*^2^ = 0.8530. *∗*Identified variables with a significant effect on the response (*p*<0.05).

**Table 3:** Experimental design and response value of path of steepest ascent.

**Run**	**Experimental value**		**Yield (** $\bar{\%}$ **)**
	*X*_3_	*X*_5_	
1	10	4	1.62
2	20	5	6.01
3	30	6	5.94
4	40	7	5.82
5	50	8	5.36

**Table 4:** Design and results of CCD.

Run	Liquid/solid ratio		Enzyme concentration		Yield of PCPP ($\bar{\%}$)
	**Code *X***_**3**_	***X***_**3**_ (mL/g)	**Code *X***_**5**_	***X***_**5**_ (%)	
1	0	20.00	0	5.00	5.97
2	-1.414	5.86	0	5.00	3.44
3	-1	10.00	1	6.00	5.00
4	0	20.00	0	5.00	5.87
5	1	30.00	-1	4.00	2.20
6	0	20.00	-1.414	3.59	0.81
7	0	20.00	0	5.00	6.1
8	0	20.00	1.414	6.41	6.01
9	0	20.00	0	5.00	6.18
10	-1	10.00	-1	4.00	1.55
11	1	30.00	1	6.00	6.89
12	0	20.00	0	5.00	6.31
13	1.414	34.14	0	5.00	5.29

**Table 5:** Results of ANOVA and regression analysis of a full second-order polynomial model for optimization of the yield of PCPP.

**Source**	**Sum of Squares**	**Mean Square**	**F Value**	***p*-value**	**significant**
**Model**	49.36	9.87	350.66	< 0.0001	*∗∗∗*

*** X***_**5**_	30.02	30.02	1066.32	< 0.0001	*∗∗∗*
***X***_**3**_	3.34	3.34	118.52	< 0.0001	*∗∗∗*
*** X***_**3**_ *** X***_**5**_	0.38	0.38	13.57	0.0078	*∗∗*
**X**_5_^2^	12.33	12.33	437.99	< 0.0001	*∗∗∗*
**X**_3_^2^	5.09	5.09	180.87	< 0.0001	*∗∗∗*
**Residual**	0.20	0.028			
**Lack of Fit**	0.077	0.026	0.86	0.5305	◆
**Pure Error**	0.12	0.030			
**Cor Total**	49.56				
**R-Squared**	0.9960				
**Adj R-Squared**	0.9932				
**Pred R-Squared**	0.9851				
**Adeq Precision**	53.58				

*∗*, *∗∗*, and *∗∗∗* represent p<0.05, p<0.01, and p<0.0001, respectively.

◆ represents “not significant”.

**Table 6:** The physicochemical properties of crude PCPP and PCPP-1a.

**Item**	***Crude PCPP***	***PCPP-1a***
**Carbohydrate (%)**	58.42 ± 5.17	80.49 ± 6.95
**Protein (%)**	1.04 ± 0.10	-
**Uronic acid (%)**	42.97 ± 3.11	21.08 ± 1.96
**Sulfuric radical (%)**	3.38 ± 0.11	1.74 ± 0.02
**Total polyphenol (mg GAE/mg dried extract)**	1.96 ± 0.18	0.44 ± 0.02
**Molecular weight (KDa)**	-	47.3
**Monosaccharide composition (%)**		
**Man**	16.61	16.59
**Rib**	5.75	7.86
**Rha**	3.86	6.83
**GlcUA**	2.68	2.11
**GalUA**	20.96	2.30
**Glc**	8.11	7.51
**Gal**	16.05	24.00
**Xyl**	14.73	18.45
**Ara**	8.20	12.36
**Fuc**	3.05	1.99

## Data Availability

The data used to support the findings of this study are available from the corresponding author upon request.
